# Menstrual and Reproductive Factors and Risk of Gastric and Colorectal Cancer in Spain

**DOI:** 10.1371/journal.pone.0164620

**Published:** 2016-10-24

**Authors:** Virginia Lope, Nerea Fernández de Larrea, Beatriz Pérez-Gómez, Vicente Martín, Victor Moreno, Laura Costas, Federico Longo, José Juan Jiménez-Moleón, Javier Llorca, Nieves Ascunce, Rosana Peiró-Pérez, Jone M. Altzibar, Adonina Tardón, Juan Alguacil, Carmen Navarro, Ángeles Sierra, Ana Belén Vega, Amaya Villafañe, Gemma Castaño-Vinyals, Manolis Kogevinas, Marina Pollán, Nuria Aragonés

**Affiliations:** 1 Cancer and Environmental Epidemiology Unit, National Center for Epidemiology, Carlos III Institute of Health, Madrid 28029, Spain; 2 Consortium for Biomedical Research in Epidemiology and Public Health (CIBER Epidemiología y Salud Pública, CIBERESP), Madrid 28029, Spain; 3 Cancer Epidemiology Research Group, Oncology and Hematology Area, IIS Puerta de Hierro (IDIPHIM), Madrid 28222, Spain; 4 Grupo de Investigación en Interacciones Gen-Ambiente y Salud, Universidad de León, León 24071, Spain; 5 IDIBELL-Catalan Institute of Oncology, L’Hospitalet de Llobregat, Barcelona 08907, Spain; 6 Department of Clinical Sciences, Faculty of Medicine, University of Barcelona, L'Hospitalet del Llobregat, Barcelona 08907, Spain; 7 Unit of Infections and Cancer, Cancer Epidemiology Research Programme, IDIBELL, Catalan Institute of Oncology, L’Hospitalet de Llobregat, Barcelona 08907, Spain; 8 Department of Medicine, University of Barcelona, L'Hospitalet del Llobregat, Barcelona 08907, Spain; 9 Medical Oncology Department, Hospital Universitario Ramón y Cajal, Madrid 28034, Spain; 10 Instituto de Investigación Biosanitaria de Granada (ibs.GRANADA)-Granada Health Research Institute (ibs.GRANADA), Granada 18012, Spain; 11 Department of Preventive Medicine and Public Health, University of Granada, Granada 18016, Spain; 12 Universidad de Cantabria-IDIVAL, Santander 39011, Spain; 13 Navarra Breast Cancer Screening Programme, Public Health Institute, Pamplona 31003, Spain; 14 Cancer and Public Health Area, Fundación Para el Fomento de la Investigación Sanitaria y Biomédica (FISABIO), Valencia 46020, Spain; 15 Public Health Division of Gipuzkoa, Donostia 20013, San Sebastián, Spain; 16 Biodonostia Research Institute, Donostia 20014, San Sebastián, Spain; 17 Instituto Universitario de Oncología, Universidad de Oviedo, Oviedo 33006, Asturias, Spain; 18 Centro de Investigación en Salud y Medio Ambiente (CYSMA), Universidad de Huelva, Huelva 21071, Spain; 19 Department of Epidemiology, Murcia Regional Health Council, IMIB-Arrixaca, Murcia 30008, Spain; 20 Department of Health and Social Sciences. Universidad de Murcia, Murcia 30003, Spain; 21 Gastroenterology, Hospital de Viladecans, Viladecans, Barcelona 08840, Spain; 22 Servicio de Cirugía, Complejo Asistencial Universitario de León, León 24071, Spain; 23 Centre for Research in Environmental Epidemiology (CREAL), Barcelona 08003, Spain; 24 IMIM (Hospital del Mar Medical Research Institute), Barcelona 08003, Spain; 25 Universitat Pompeu Fabra (UPF), Barcelona 08003, Spain; National Cancer Center, JAPAN

## Abstract

**Background:**

Sex hormones play a role in gastric cancer and colorectal cancer etiology, however, epidemiological evidence is inconsistent. This study examines the influence of menstrual and reproductive factors over the risk of both tumors.

**Methods:**

In this case-control study 128 women with gastric cancer and 1293 controls, as well as 562 female and colorectal cancer cases and 1605 controls were recruited in 9 and 11 Spanish provinces, respectively. Population controls were frequency matched to cases by age and province. Demographic and reproductive data were directly surveyed by trained staff. The association with gastric, colon and rectal cancer was assessed using logistic and multinomial mixed regression models.

**Results:**

Our results show an inverse association of age at first birth with gastric cancer risk (five-year trend: OR = 0.69; p-value = 0.006). Ever users of hormonal contraception presented a decreased risk of gastric (OR = 0.42; 95%CI = 0.26–0.69), colon (OR = 0.64; 95%CI = 0.48–0.86) and rectal cancer (OR = 0.61; 95%CI = 0.43–0.88). Postmenopausal women who used hormone replacement therapy showed a decreased risk of colon and rectal tumors. A significant interaction of educational level with parity and months of first child lactation was also observed.

**Conclusion:**

These findings suggest a protective role of exogenous hormones in gastric and colorectal cancer risk. The role of endogenous hormones remains unclear.

## Introduction

Colorectal cancer (CRC) in Spain, with an estimated 16071 new cases in 2014 [1], represents the second most common tumor in women and the second leading cause of death, accounting for 15.3% of all female cancer-related deaths in 2013 [2]. In Spain, as in other developed countries [3], there has been an increase in incidence due to this type of cancer, slightly attenuated around 1995 [4]. However, mortality rates have reached a plateau since the beginning of this century [5].

Gastric cancer (GC) in Spanish women occupies the tenth position in incidence, with an adjusted rate of 7.5 cases per 100,000 estimated for 2012 [6]. In terms of mortality, the rate estimated for this same year was 4.8 per 100,000, accounting for 5.3% of all female cancer-related deaths in 2013 [2]. The small difference between the incidence and mortality rates is due to the low survival recorded for this tumor, which is estimated in Spain to be 26.0% at 5 years [1].

Hormonal factors may play a role in the etiology of both tumors: incidence is approximately twofold higher among males than among females [6], even though differential exposure to established risk factors cannot totally explain these differences. On the other hand, it has been suggested that estrogens may offer protection against the development of both tumors, and this protective effect seems to be modulated through estrogen receptors (ER) identified in non-cancerous and cancerous gastric [7] and colonic tissue [8, 9]. Recent studies indicate that the protection conferred to CRC risk could be limited to certain molecular tumor subtypes [8] and to exogenous estrogens exposure [9, 10]. On the contrary, while some studies have observed lower GC risk associated with the exposure to estrogens of both ovarian and exogenous origin [11], other authors have detected this association with endogenous estrogen exposure only [12].

The role of menstrual and reproductive factors in the etiology of GC, and mainly CRC, has drawn interest in the literature, but findings are not consistent, especially in the case of GC where most studies have been limited by small number of cases. This study sought to investigate the influence of menstrual and reproductive factors on female GC and CRC risk in a large population-based case control study in Spain. We also assessed if these associations differ by specific CRC subsite. Finally, taking into account that reproductive patterns and the use of hormonal compounds are strongly influenced by womens’ educational level [13] and that highly educated women tend to have healthier life-styles [14, 15], we evaluated the influence of reproductive factors separately in women with high and low educational background.

## Materials and Methods

### Study population

Multicase-control Spain study (MCC-Spain, www.mccspain.org) [16], with population controls and incident cases was carried out between September 2008 and December 2013 to investigate the influence of environmental factors and their interaction with genetic factors in highly prevalent tumors or with peculiar epidemiological characteristics in Spain. Cases of breast, prostate, gastric, colorectal tumors and chronic lymphocytic leukemias were recruited from 23 hospitals in 12 Spanish provinces. Inclusion criteria required that participants should have resided for at least 6 months in the study areas, had to be aged 20–85 and had to be mentally qualified to answer the questionnaire. A group of controls, common for the five types of tumors, was randomly selected from the administrative records of a number of primary care health centers within the catchment areas of the hospitals where cases were recruited. We made an initial estimate of the age-sex distribution that cases–all combined- would have in each region, according to the tumors they recruited and to the cancer incidence rates from Spanish cancer registries. We applied these estimates to predefine the age-sex distribution of our population-based controls, which were selected randomly from the general practitioner lists of each hospital catchment area. When the recruitment of cases ended, we compared again the age- sex- distribution of cases and controls and recruited new participants if needed in an attempt to ensure that each case had at least one control of the same 5-year age interval and sex in each region. Controls were initially contacted via telephone and those who agreed to participate in the study were scheduled for a personal interview. Cases of GC were recruited in Madrid, Barcelona, León, Navarra, Cantabria, Asturias, Huelva, Valencia and Granada. CRC cases were also recruited in Guipúzcoa and Murcia. Our research personnel actively searched for new cases, through regular visits to the collaborating hospital departments (gastroenterology, oncology, general surgery, radiotherapy and pathology) and reviewed the hospital admission registries weekly.

**Fig 1:**
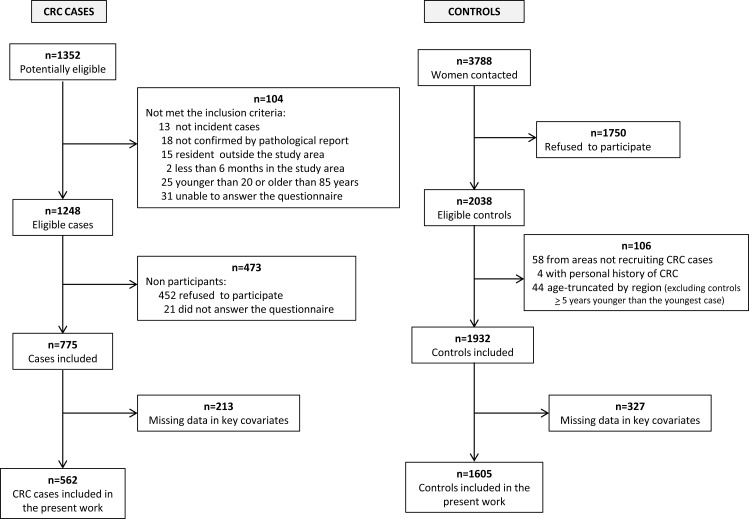
Flow chart displaying the selection process of colorectal cancer cases and controls. MCC-Spain study 2008–2013.

**Fig 2:**
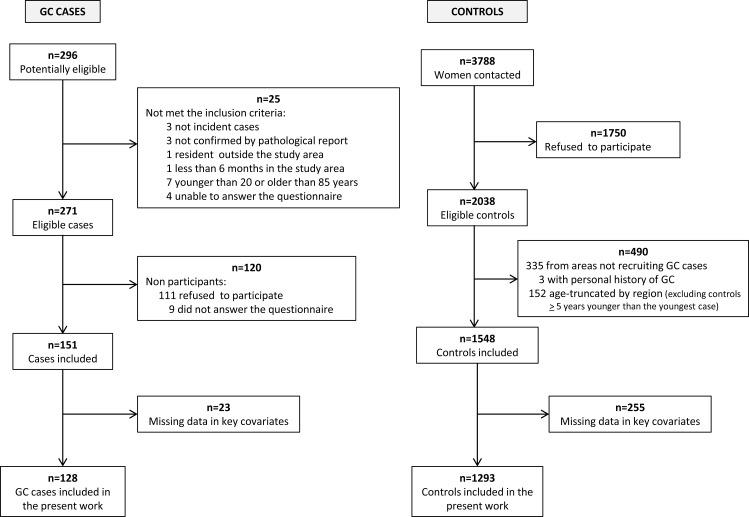
Flow chart displaying the selection process of gastric cancer cases and controls. MCC-Spain study 2008–2013.

For the present study we recruited a total of 151 histologically-confirmed female GC cases (codes C16:*Malignant neoplasm of stomach* and D00.2:*Carcinoma in situ of stomach*, according to the 10th revision of the International Statistical Classification of Diseases), and 775 female CRC cases (codes C18:*Malignant neoplasm of colon*, C19:*Malignant neoplasm of rectosigmoid junction*, C20:*Malignant neoplasm of rectum*, D01.0:C*arcinoma in situ of colon*, D01.1:C*arcinoma in situ of rectosigmoid junction* and D01.2:*Carcinoma in situ of rectum*) with no prior history of the disease and diagnosed within the recruitment period. Those controls with a history of GC or CRC were excluded, as well as those who resided in provinces that had not recruited these tumors and those that were more than five years younger than the youngest case included in each region. A total of 1548 and 1932 female controls were included for the GC and CRC analyses, respectively. Flow charts displaying the selection process of CRC cases and controls and GC cases and control are shown in Fig 1 and Fig 2 respectively.

### Ethical approval

The protocol of MCC-Spain was approved by Ethics Committees of the participating institutions: Comité Ético de Investigación Clínica (CEIC) del Instituto Municipal de Asistencia Sanitaria de Barcelona; CEIC del Hospital Universitario de Bellvitge; CEIC de Navarra; CEIC del Hospital Universitario La Paz; CEIC del Hospital Universitario Ramón y Cajal; CEIC de Cantabria; CEIC de la Dirección General de Salud Pública y Centro Superior de Investigación en Salud Pública; CEIC del Hospital General Universitario Jose Mª Morales Meseguer; Comité de Ética de la Investigación de la Provincia de Huelva; CEIC de León; Comité Ético de Investigación del Principado de Asturias; Comité de Ética de la Investigación Biomédica Provincial de Granada; Comité de Ética en Investigación Humana de la Universidad de Granada; Comité Ético de Investigación de la Comunidad Autónoma del País Vasco. All participants were informed about the study objectives and signed an informed consent. Confidentiality of data is secured removing personal identifiers in the datasets. The database was registered in the Spanish Agency for Data Protection, number 2102672171.

### Data collection

Exposure information was collected by trained interviewers through face-to-face interviews using a structured electronic questionnaire, including detailed information on demographic factors, occupation, personal and family history, lifestyle and diet [17]. Menstrual factors gathered by the questionnaire included age at menarche, regularity of the menstrual cycle, menopausal status, age and cause of menopause, use of hormonal contraceptives and hormone replacement therapy. Regarding reproductive history, the questionnaire collected information on fertility problems and their treatment, number of miscarriages, number of children, newborns' sex, year of birth, gestational age and duration of maternal lactation.

### Statistical analyses

Descriptive analyses of participants’ characteristics were done for GC, colon cancer (CC) and rectal cancer (RC) cases and controls. We used absolute figures and percentages to describe categorical variables, and means and standard deviations to describe continuous variables. Significant differences between cases and controls were tested using Pearson chi-square and Student's t-test for categorical and continuous variables respectively.

The association of menstrual and reproductive variables with GC, CC and RC was assessed using logistic mixed regression models, including the province as a random effect term to account for unexplained heterogeneity associated with province, such as distribution of unmeasured potential risk factors or differences between interviewers. Models were adjusted for age, educational level, body mass index (BMI) one year prior to the interview, tobacco consumption, family history of the studied cancer, hormone replacement therapy use and hormonal contraception use. We also conducted an additional analysis in CRC cases and controls stratifying by educational level, not done for GC due to the low number of cases. Finally, a sensitivity analysis was done additionally adjusting for calorie intake, red meat, processed meat, fruits, vegetables and alcohol consumption in those participants who fulfilled the food frequency questionnaire (Supporting Information).

CRC patients were classified according to tumor subsite: CC and RC (two cases were of unspecified subsite). Since our response variable has three categories (0 = controls, 1 = CC cases and 2 = RC cases), we fitted multinomial logistic regression models to evaluate the association of menstrual and reproductive factors with the above-mentioned CRC subsites. These models were adjusted by the same set of variables described above, including the province as a random effect term. Heterogeneity of effects was tested using a Wald test to compare the coefficients obtained for the different CRC subsites. Data were analyzed using STATA/MP 13.1 software.

## Results

Response rates were 53.8% for healthy female controls, 62.1% for female CRC cases and 55.7% for female GC cases. Results presented in this manuscript are based on women with no missing values in any of the selected confounders (73% of CRC cases, 85% of GC cases and 83% of controls). We included 562 CRC cases (364 colon cases and 196 rectal cases) and 1605 controls, as well as 128 GC cases and 1293 controls. Table 1 shows the main characteristics of these women. In general, gastric, colon and rectal cases were older and had higher BMI than controls. They also smoked less, had lower educational level, had a delayed menarche, had more children, had their first child at a younger age, had longer breastfeeding periods and reported to use less hormone replacement therapy and hormonal contraception than controls.

**Table 1:** Socio-demographic, menstrual and reproductive characteristics in gastric and colorectal cancer cases and controls.

		GASTRIC CANCER					COLORECTAL CANCER							
		Controls		Cases			Controls		Colon cases			Rectal cases		
		(N = 1293)		(N = 128)			(N = 1605)		(N = 364)			(N = 196)		
		N	(%)	N	(%)	p-val	N	(%)	N	(%)	p-val	N	(%)	p-val
Age, mean(SD)		59.5	(12.6)	65.0	(14.2)	<0.001	58.9	(12.5)	65.5	(11.4)	<0.001	64.3	(11.8)	<0.001
Educational level						<0.001					<0.001			<0.001
	Less than primary school	197	(15%)	52	(41%)		259	(16%)	108	(30%)		65	(33%)	
	Primary school completed	422	(33%)	47	(37%)		490	(31%)	140	(38%)		74	(38%)	
	Secondary school	392	(30%)	24	(19%)		495	(31%)	73	(20%)		35	(18%)	
	University graduate	282	(22%)	5	(4%)		361	(22%)	43	(12%)		22	(11%)	
BMI						0.003					<0.001			0.045
	<20 Kg/m^2^	91	(7%)	5	(4%)		111	(7%)	15	(4%)		13	(7%)	
	20–24 Kg/m^2^	542	(42%)	36	(28%)		681	(42%)	116	(32%)		64	(33%)	
	25–29 Kg/m^2^	409	(32%)	50	(39%)		506	(32%)	139	(38%)		78	(40%)	
	>29 Kg/m^2^	251	(19%)	37	(29%)		307	(19%)	94	(26%)		41	(21%)	
Smoking						0.001					<0.001			0.004
	Never smoker	778	(60%)	97	(76%)		934	(58%)	257	(71%)		135	(69%)	
	Former smoker > 1.5 years	257	(20%)	10	(8%)		332	(21%)	56	(15%)		38	(19%)	
	Smoker or former smoker <1.5 years	258	(20%)	21	(16%)		339	(21%)	51	(14%)		23	(12%)	
Family history colorectal/gastric cancer						<0.001					<0.001			0.005
	None	1160	(90%)	90	(70%)		1397	(87%)	283	(78%)		160	(82%)	
	Second degree only	77	(6%)	9	(7%)		67	(4%)	15	(4%)		7	(4%)	
	1 first degree	53	(4%)	20	(16%)		129	(8%)	55	(15%)		23	(12%)	
	>1 first degree	3	(0%)	9	(7%)		12	(1%)	11	(3%)		6	(3%)	
Age at menarche, mean(SD)		12.8	(1.7)	13.2	(2.0)	0.018	12.8	(1.6)	13.0	(1.7)	0.024	13.2	(1.6)	0.006
Number of children						0.006					0.001			0.047
	None	235	(18%)	22	(17%)		278	(17%)	43	(12%)		27	(14%)	
	1–2	716	(55%)	55	(43%)		902	(56%)	190	(52%)		99	(51%)	
	3–4	279	(22%)	39	(30%)		350	(22%)	103	(28%)		58	(30%)	
	>4	61	(5%)	12	(9%)		72	(4%)	28	(8%)		12	(6%)	
Age at first birth, mean(SD)		26.7	(4.8)	24.8	(4.7)	<0.001	26.8	(4.8)	26.2	(4.5)	0.040	26	(5.2)	0.051
Lactation first child (months), mean(SD)		4.5	(5.7)	5.5	(5.8)	0.113	4.4	(5.6)	5.4	(6.1)	0.008	5.6	(6.1)	0.020
History of miscarriages						0.768					0.013			0.597
	None	985	(76%)	99	(77%)		1231	(77%)	301	(83%)		147	(75%)	
	One or more	308	(24%)	29	(23%)		374	(23%)	63	(17%)		49	(25%)	
Menopausal status						0.001					<0.001			<0.001
	Premenopausal	340	(26%)	17	(13%)		456	(28%)	39	(11%)		29	(15%)	
	Posmenopausal	953	(74%)	111	(87%)		1149	(72%)	325	(89%)		167	(85%)	
Hormonal contraception use						<0.001					<0.001			<0.001
	Never	668	(52%)	101	(79%)		809	(50%)	254	(70%)		135	(69%)	
	Ever	625	(48%)	27	(21%)		796	(50%)	110	(30%)		61	(31%)	
Postmenopausal women														
Hormone therapy use						0.121					<0.001			0.016
	Never	858	(90%)	105	(95%)		1025	(89%)	311	(96%)		159	(95%)	
	Ever	95	(10%)	6	(5%)		124	(11%)	14	(4%)		8	(5%)	
Age at menopause, mean(SD)		48.4	(5.3)	47.9	(5.6)	0.397	48.6	(5.3)	48.7	(5.2)	0.667	48.7	(5.0)	0.663
Fertility time (years), mean(SD)		35.5	(5.4)	34.7	(5.7)	0.183	35.7	(5.4)	35.7	(5.2)	0.949	35.5	(4.9)	0.638

Table 2 shows the association between GC, CC and RC risk and menstrual and reproductive factors. Age at first birth displayed an inverse association with GC, decreasing the risk by 31% for every five years increase in age at first birth (P = 0.006) (>29 years vs. <25: OR = 0.52; 95%CI = 0.28–0.97). This result remained significant even after adjustment for number of children (five-year trend: OR = 0.71; P = 0.014) or additionally adjusting for calorie intake, red meat, processed meat, fruits, vegetables and alcohol consumption (five-year trend: OR = 0.71; P = 0.046) (S1 Table). Women who ever used hormonal contraception showed a decreased gastric (OR = 0.42; 95%CI = 0.26–0.69), colon (OR = 0.64; 95%CI = 0.48–0.86), and rectal (OR = 0.61; 95%CI = 0.43–0.88) cancer risk, results that are confirmed after adjustment for calorie intake, red meat, processed meat, fruits, vegetables and alcohol consumption. The protection conferred by hormonal contraception use was stronger for those who used it more than 5 years for all cancer types (OR for GC = 0.26; 95%CI = 0.10–0.67; OR for CC = 0.59; 95%CI = 0.38–0.92; OR for RC = 0.53; 95%CI = 0.30–0.95). Finally, postmenopausal women who ever used hormone replacement therapy showed a decreased CC risk (OR = 0.44; 95%CI = 0.24–0.78) and almost significant decreased RC risk (OR = 0.51; 95%CI = 0.24–1.07). No significant differences were detected between colon and rectal tumors in any of the variables analyzed. Also, no significant associations were observed between GC, CC and RC risk and other menstrual and reproductive variables (history of miscarriages, age at menopause, fertility time, fertility time without children, time since last child, time since menopause, and each woman's cumulative lifetime lactation) (data not shown).

**Table 2:** Association between menstrual and reproductive characteristics and gastric and colorectal cancer risk.

			GASTRIC CANCER						COLORECTAL CANCER													
										COLON CANCER						RECTAL CANCER						
Variable^a^		controls	cases	OR^b^	95% CI			p-val	controls	cases	OR^b^	95% CI			p-val	cases	OR^b^	95% CI			p-val	P-int.^c^
Premenopausal status		340	17	0.94	0.45	-	2.00	0.882	456	39	0.65	0.41	-	1.03	0.068	29	0.82	0.47	-	1.45	0.500	0.494
Nulliparity		235	22	1.12	0.65	-	1.92	0.682	278	43	0.78	0.53	-	1.13	0.184	27	0.95	0.60	-	1.50	0.824	0.464
Parous women																						
	Age at first birth (years)																					
	<25	369	58	1.00					437	126	1.00					80	1.00					
	25–29	409	31	0.52	0.32	-	0.86	0.010	533	129	0.84	0.63	-	1.13	0.242	58	0.62	0.43	-	0.90	0.013	
	>29	273	16	0.52	0.28	-	0.97	0.040	346	64	0.79	0.55	-	1.14	0.207	30	0.63	0.39	-	1.00	0.052	
	Five-year trend*			*0*.*69*	*0*.*53*	*-*	*0*.*90*	*0*.*006*			*0*.*95*	*0*.*82*		*1*.*10*	*0*.*473*		*0*.*95*	*0*.*78*		*1*.*14*	*0*.*556*	0.978
	No. of children																					
	1–2	716	55	1.00					902	190	1.00					99	1.00					
	3–4	279	39	1.27	0.78	-	2.04	0.337	350	103	0.96	0.72	-	1.29	0.810	58	1.11	0.77	-	1.62	0.574	
	> 4	61	12	1.77	0.82	-	3.82	0.144	72	28	1.09	0.66	-	1.80	0.743	12	0.99	0.50	-	1.96	0.977	
	Trend per child*			*1*.*13*	*0*.*96*	*-*	*1*.*33*	*0*.*145*			*1*.*04*	*0*.*94*		*1*.*15*	*0*.*454*		*0*.*97*	*0*.*85*		*1*.*11*	*0*.*703*	0.394
	Lactation first child (months)																					
	None	185	23	1.40	0.80	-	2.48	0.241	241	58	1.04	0.72	-	1.48	0.841	34	1.29	0.82	-	2.02	0.274	
	1–6	542	43	1.00					682	141	1.00					67	1.00					
	>6	177	28	1.53	0.89	-	2.65	0.126	217	80	1.26	0.89	-	1.77	0.192	44	1.43	0.93	-	2.21	0.105	
	Six-month trend*			*1*.*10*	*0*.*89*	*-*	*1*.*35*	*0*.*385*			*1*.*10*	*0*.*95*		*1*.*26*	*0*.*199*		*1*.*09*	*0*.*92*		*1*.*30*	*0*.*294*	0.987
No. of miscarriages																						
	None	985	99	1.00					1231	301	1.00					147	1.00					
	One or more	308	29	1.21	0.76	-	1.93	0.416	374	63	0.75	0.55	-	1.03	0.073	49	1.23	0.86	-	1.76	0.246	0.089
Age at menarche (years)																						
	<12	254	24	1.25	0.72	-	2.16	0.430	320	70	1.15	0.83	-	1.61	0.400	33	0.94	0.61	-	1.46	0.791	
	12–13	599	46	1.00					749	144	1.00					82	1.00					
	>13	433	56	1.26	0.81	-	1.96	0.298	523	146	1.17	0.89	-	1.53	0.267	80	1.15	0.81	-	1.61	0.438	
	Trend per year*			*1*.*05*	*0*.*94*	*-*	*1*.*17*	*0*.*397*			*1*.*01*	*0*.*94*		*1*.*09*	*0*.*713*		*1*.*07*	*0*.*97*	*-*	*1*.*17*	*0*.*155*	0.319
Hormonal contraception use																						
	Never	668	101	1.00					809	254	1.00					135	1.00					
	Ever	625	27	0.42	0.26	-	0.69	0.001	796	110	0.64	0.48	-	0.86	0.003	61	0.61	0.43	-	0.88	0.009	0.820
	< = 5 years	255	16	0.62	0.34	-	1.13	0.121	315	47	0.69	0.48	-	1.00	0.052	27	0.68	0.42	-	1.09	0.108	
	>5 years	191	5	0.26	0.10	-	0.67	0.006	249	30	0.59	0.38	-	0.92	0.020	16	0.53	0.30	-	0.95	0.034	
	Not known	179	6	0.31	0.13	-	0.75	0.009	232	33	0.63	0.41	-	0.97	0.036	18	0.60	0.35	-	1.04	0.069	
Postmenopausal women																						
Age at menopause (years)																						
	< = 45	208	17	0.68	0.35	-	1.33	0.260	245	57	0.81	0.55	-	1.19	0.283	40	1.15	0.72	-	1.84	0.567	
	46–49	226	31	1.07	0.60	-	1.92	0.821	259	78	1.02	0.71	-	1.47	0.902	32	0.87	0.53	-	1.44	0.595	
	50–52	238	31	1.00					294	90	1.00					45	1.00					
	>52	167	15	0.70	0.36	-	1.39	0.312	212	64	0.92	0.63	-	1.34	0.661	29	0.83	0.50	-	1.38	0.464	
	Five-year trend*			*0*.*92*	*0*.*75*	*-*	*1*.*14*	*0*.*451*			*1*.*00*	*0*.*87*	*-*	*1*.*13*	*0*.*947*		*0*.*99*	*0*.*83*	*-*	*1*.*18*	*0*.*910*	0.955
Fertility time (years)																						
	<33	202	28	1.00					236	73	1.00					36	1.00					
	33–36	233	23	0.75	0.40	-	1.41	0.371	269	82	0.98	0.67	-	1.43	0.916	46	1.09	0.68	-	1.77	0.715	
	37–39	213	22	0.75	0.40	-	1.43	0.386	263	69	0.83	0.56	-	1.22	0.340	33	0.79	0.47	-	1.32	0.366	
	>39	185	19	0.82	0.43	-	1.59	0.565	233	64	0.82	0.55	-	1.23	0.340	30	0.77	0.45	-	1.31	0.333	
	Five-year trend*			*0*.*91*	*0*.*75*	*-*	*1*.*12*	*0*.*379*			*0*.*99*	*0*.*87*	*-*	*1*.*12*	*0*.*871*		*0*.*94*	*0*.*80*	*-*	*1*.*11*	*0*.*463*	0.588
Hormone therapy use																						
	Never	858	105	1.00					1025	311	1.00					159	1.00					
	Ever	95	6	0.63	0.26	-	1.53	0.306	124	14	0.44	0.24	-	0.78	0.005	8	0.51	0.24	-	1.07	0.075	0.743

^a^ Totals do not add up because of missing values.

^b^ Odds ratios (ORs) and 95% confidence intervals (95% CI) adjusted for age, educational level, BMI 1-year prior to the interview, family history of gastric/colorectal cancer, tobacco, hormone therapy use and hormonal contraception use (the latter two variables were excluded as confounders when analyzing their association with gastric and colorectal cancer risk). Province was included as a random effect term.

^c^ P-int.: P value of the interaction term between tumor subsite and the corresponding variable.

* In italics: ORs, 95% CI and P values obtained with the corresponding variable as a continuous term.

Table 3 presents the results of these associations with CRC risk stratified by educational background. Number of children and months of lactation displayed different effects in women with low and high educational level, with the interaction term proving statistically significant (P = 0.001 and 0.040 respectively): while parity showed a positive trend among women with low educational level (primary school or less), in women with higher level of education (secondary school or university) the OR decreased by 23% for every child (P = 0.012). With respect to months of lactation, whereas those women with lower educational background who breastfed their first child for longer periods registered an increased CRC risk (>6 months vs. 1–6 months: OR = 1.55; 95%CI = 1.10–2.18; P trend = 0.031), those with higher education, showed the opposite effect, although this association was not statistically significant, probably due to the small number of cases who breastfed more than six months. The protection conferred by hormonal contraception and hormone replacement therapy use was in evidence in both groups, although it was stronger for women with low educational level. While duration of hormonal contraception use was not associated with CRC risk in women with low educational level, in those with higher educational background the risk decreased by 28% for every 5-year increase in hormonal contraception use (P = 0.032) (data not shown). Finally we also detected a U-shape association with age at menarche among women with higher educational level (<12 years vs. 12–13: OR = 1.51; 95%CI = 0.98–2.33; >13 years vs. 12–13: OR = 1.57; 95%CI = 1.06–2.34). These analyses were repeated additionally adjusting for the above mentioned dietary variables, but this led to no change in the results (S2 Table).

**Table 3:** Association between menstrual and reproductive characteristics and colorectal cancer risk by educational level.

		COLORECTAL CANCER														
		Primary school or less							Secondary school or University							
Variable^a^		controls	cases	OR^b^	95% CI			p-val	controls	cases	OR^b^	95% CI			p-val	P-int.^c^
Premenopausal status		99	27	0.86	0.51	-	1.46	0.575	357	42	0.65	0.42	-	1.01	0.054	0.360
Nulliparity		70	29	0.71	0.44	-	1.15	0.167	208	42	0.95	0.64	-	1.41	0.798	0.366
Parous women																
	Age at first birth (years)															
	<25	277	164	1.00					160	42	1.00					
	25–29	276	136	0.77	0.57	-	1.04	0.091	257	52	0.72	0.45	-	1.15	0.168	
	>29	117	57	0.75	0.51	-	1.11	0.152	229	37	0.68	0.41	-	1.13	0.138	
	Five-year trend*			*0*.*97*	*0*.*83*	*-*	*1*.*13*	*0*.*671*			*0*.*91*	*0*.*74*	*-*	*1*.*11*	*0*.*353*	0.629
	No. of children															
	1–2	420	184	1.00					482	106	1.00					
	3–4	212	139	1.27	0.94	-	1.70	0.116	138	22	0.54	0.32	-	0.90	0.019	
	> 4	46	35	1.33	0.81	-	2.20	0.262	26	5	0.49	0.18	-	1.38	0.180	
	Trend per child*			*1*.*10*	*0*.*99*	*-*	*1*.*21*	*0*.*072*			*0*.*77*	*0*.*63*	*-*	*0*.*94*	*0*.*012*	0.001
	Lactation first child (months)															
	None	137	65	1.12	0.77	-	1.63	0.548	104	27	1.13	0.68	-	1.88	0.646	
	1–6	323	134	1.00					359	75	1.00					
	>6	139	112	1.55	1.10	-	2.18	0.012	78	12	0.68	0.35	-	1.34	0.266	
	Six-month trend*			*1*.*17*	*1*.*01*	*-*	*1*.*34*	*0*.*031*			*0*.*82*	*0*.*59*	*-*	*1*.*15*	*0*.*249*	0.040
No. of miscarriages																
	None	584	317	1.00					647	132	1.00					
	One or more	165	70	0.81	0.59	-	1.13	0.212	209	43	1.09	0.74	-	1.62	0.657	0.686
Age at menarche (years)																
	<12	134	58	0.84	0.57	-	1.22	0.353	186	45	1.51	0.98	-	2.33	0.059	
	12–13	317	161	1.00					432	66	1.00					
	>13	289	166	0.99	0.75	-	1.32	0.972	234	61	1.57	1.06	-	2.34	0.026	
	Trend per year*			*1*.*03*	*0*.*96*	*-*	*1*.*11*	*0*.*430*			*1*.*04*	*0*.*94*	*-*	*1*.*16*	*0*.*444*	0.861
Hormonal contraception use																
	Never	475	305	1.00					334	85	1.00					
	Ever	274	82	0.55	0.40	-	0.75	<0.001	522	90	0.75	0.53	-	1.06	0.099	0.179
	< = 5 years	105	34	0.59	0.38	-	0.92	0.019	210	41	0.82	0.53	-	1.26	0.368	
	>5 years	78	25	0.66	0.40	-	1.10	0.111	171	21	0.51	0.30	-	0.87	0.014	
	Not known	91	23	0.42	0.25	-	0.70	0.001	141	28	0.94	0.57	-	1.55	0.806	
Postmenopausal women																
Age at menopause (years)																
	< = 45	142	64	0.80	0.54	-	1.20	0.283	103	33	1.22	0.69	-	2.15	0.494	
	46–49	143	79	0.95	0.65	-	1.39	0.787	116	31	1.07	0.60	-	1.90	0.815	
	50–52	164	102	1.00					130	33	1.00					
	>52	138	74	0.88	0.60	-	1.30	0.528	74	20	0.91	0.48	-	1.73	0.774	
	Five-year trend*			*1*.*05*	*0*.*92*	*-*	*1*.*21*	*0*.*465*			*0*.*87*	*0*.*71*	*-*	*1*.*06*	*0*.*161*	0.114
Fertility time (years)																
	<33	137	79	1.00					99	30	1.00					
	33–36	162	93	1.00	0.67	-	1.48	0.992	107	35	1.04	0.58	-	1.85	0.904	
	37–39	144	74	0.85	0.56	-	1.28	0.428	119	28	0.74	0.40	-	1.34	0.317	
	>39	138	72	0.86	0.57	-	1.30	0.477	95	23	0.68	0.36	-	1.28	0.231	
	Five-year trend*			*1*.*02*	*0*.*89*	*-*	*1*.*16*	*0*.*774*			*0*.*87*	*0*.*71*	*-*	*1*.*06*	*0*.*168*	0.189
Hormone therapy use																
	Never	585	350	1.00					440	121	1.00					
	Ever	65	10	0.30	0.15	-	0.60	0.001	59	12	0.74	0.38	-	1.47	0.393	0.064

^a^ Totals do not add up because of missing values.

^b^ Odds ratios (ORs) and 95% confidence intervals (95% CI) adjusted for age, BMI 1-year prior to the interview, family history of colorectal cancer, tobacco, hormone therapy use and hormonal contraception use (the latter two variables were excluded as confounders when analyzing their association with colorectal cancer risk). Province was included as a random effect term.

^c^ P-int.: P value of the interaction term between educational level and the corresponding variable.

* In italics: ORs, 95% CI and P values obtained with the corresponding variable as a continuous term.

## Discussion

This case-control study with population controls examines the association between recalled menstrual and reproductive factors and GC and CRC risk, and evaluates whether the effect differ by educational level. Our results show a decreased GC risk associated with older age at first birth; a decreased GC, CC and RC risk associated with ever use of hormonal contraception and a decreased CC and RC risk among postmenopausal hormone therapy users. Women with low educational level who had more children or who breastfed their first child for longer periods registered an increased CRC risk, while those with higher educational background showed the opposite effect.

One of the main strengths of this study is its large sample size, since to date it is the largest epidemiological study that analyses the association between GC and CRC risk and menstrual and reproductive factors in the Spanish population. The study was carried out in 11 Spanish provinces, covering rural and urban areas. In addition, we have used histologically confirmed incident cases and population-based controls. Finally, the random province-specific intercept term included in our statistical models allowed us to take into account unexplained heterogeneity due to unmeasured factors across different provinces.

Some limitations should also be considered. First, self-reported information is subjected to recall bias. However, if this bias exists, it would probably be non-differential, since the possible association between reproductive factors and GC and CRC risk is largely unknown. Several previous studies have concluded that self-reported information of age at menopause, age at menarche, number of children, age at first pregnancy, age at first and last birth and spontaneous abortions are recalled with reasonable accuracy [18, 19]. These same variables were used in a previous study of our group that analyzed the influence of obstetric factors on mammographic density in adult Spanish women [20]. In a quality control analysis of that study, which included re-test in a subsample of women, we showed acceptable levels of reproducibility. Regarding a potential selection bias, we have evaluated possible differences between women with complete and missing data in terms of demographic and other confounding variables and we observed that women unable to provide this information were older and had lower educational background than those who did, being this difference similar in cases and controls. Another limitation is the high number of missing values in dietary variables. However, when we replicated the analyses introducing these variables in the models (S1 Table and S2 Table) results were not altered. It could be possible that unmeasured confounders affect our findings. Nevertheless, most established GC risk factors were controlled in the present study, with the exception of *Helicobacter pylori* infection. However, the infection caused by this bacterium is not a confounding factor in our study since, in agreement with the literature, no association was observed between this infection, measured by a serological assay, and the reproductive factors studied here (data not shown). Moreover, those unmeasured characteristics that could have a geographical distribution have been at least partly accounted for through the random effect province term included in our statistical analyses. Finally, we have small sample size to find statistically significant associations when evaluating certain subgroup associations for GC, such as the stratified analyses by anatomic subsite, histologic type or educational level.

Age at first birth was inversely associated with GC risk in the present study. In a previous meta-analysis no association with this variable was detected [11]. Nevertheless, some studies of this meta-analysis have found a borderline inverse association of age at first birth with GC in general [21], with all subsites and histological types of GC [22], with adenocarcinomas of the gastric cardia [23, 24], and with non-cardia gastric cancer in postmenopausal women[23].

Our results suggest a protective role of exogenous female hormones on gastric and colorectal cancer risk. Previous studies of oral contraceptives and GC have reported risk estimates from 0.79 to 2.50, with a pooled relative risk for ever use of 1.11 [11]. Only Frise et al detected a no significant decreased risk of gastric adenocarcinoma, more pronounced for the intestinal histologic subtype [25]. Nevertheless, the protective effect of hormone replacement therapy on GC risk has been reported in several studies [11, 26]. Evidence suggests that estrogens may offer protection against the development and progression of GC by acting on ERα and ERβ. The biological pathway is unclear, but several mechanisms have been suggested, such as the increased expression of trefoil factor genes, the inhibition of oncogenes’ expression or decreasing bile acid concentration [27]. For this reason, it would be reasonable to think that parity might be associated with GC risk through these pathways. However, in consonance with our findings, the most recent meta-analysis of prospective cohort studies found no significant association [28].

With respect to CRC, three previous meta-analyses described an inverse association with oral contraceptives use, with summary relative risks of 0.81 [29] and 0.82 [30, 31]. Two of them detected no differences according to duration of use, although there were indications that the protection was stronger for more recent use [29, 30]. Meanwhile, Luan et al described a statistically significant nonlinear inverse association with duration of use [31]. Regarding hormone replacement therapy, two previous meta-analyses found an approximately 20% reduction in CRC risk among ever users [32, 33], similar for colon and rectal tumors [32]. This protective effect seems to be modulated through the ERβ, the predominant ER in the colon. During the tumorigenesis process, the ERβ expression in colonocytes is lost, and hormone replacement therapy exerts its effects through preventing this loss [8]. Increased local concentration of estrogens reduces the production of carcinogenic secondary bile acid, limits DNA damage and microsatellite instability and inhibits cell proliferation of colonic tumors [9].

When analyzing CRC risk, we have detected an interaction between months of first birth lactation and parity with educational level. Frise et al, in a study of reproductive factors and risk of gastric adenocarcinoma, also detected a statistically significant interaction between income level and parity [25]. With respect to lactation, previous studies have detected no association [34–36], except Lo et al, who found an inverse association with CRC risk [37]. Regarding parity, Guan et al, in a meta-analysis of prospective studies, found a 5% decreased CRC risk among parous versus nulliparous women, although they found no dose-response effect [38]. La Vecchia et al, in 6 out of 18 studies found significant protection by parity on CC or CRC risk [39]. When we additionally adjusted by other dietary CRC risk factors (such as calorie intake, red meat, processed meat, fruits, vegetables, and alcohol consumption) we also observed a significant interaction term with parity (P<0.001) and almost significant with duration of lactation (P = 0.051). Since we do not find a clear biologic rationale for this result, it should be interpreted with caution in terms of prevention. It is possible that we were unable to completely adjust for these imprecisely measured factors, which may have led to some residual confounding. Other lifestyle conditions not yet identified could have influenced on these associations, as well as early-life conditions that could not be controlled for, given that these women grew up in a tough era marked by the Spanish civil war and long postwar period.

In brief, our results provide some support for the hypothesis that oral contraceptives and hormone replacement therapy decrease GC and CRC risk in women. We detected a decreased GC risk associated with advanced maternal age at first birth and a significant interaction of educational level with parity and breastfeeding. These findings are consistent with the exogenous hormone hypothesis. However, the role of endogenous hormones on GC and CRC risk remains to be elucidated, and should be explored taking into account socioeconomic factors and lifestyles closely related with reproductive patterns.

## Supporting Information

S1 Table Association between menstrual and reproductive characteristics and gastric and colorectal cancer risk, additionally adjusted for dietary factors. (DOCX)

S2 Table Association between menstrual and reproductive characteristics and colorectal cancer risk by educational level, additionally adjusted for dietary factors. (DOCX)
