# Supplementary material for: Menstrual and Reproductive Factors and Risk of Gastric and Colorectal Cancer in Spain
Source: PLoS One. 2016 Oct 24;11(10):e0164620. doi: 10.1371/journal.pone.0164620 (PMC5077095; doi:10.1371/journal.pone.0164620)
Supplement: S1 Table — (DOCX) [file pone.0164620.s001.docx]

**Supplementary Table 1.** Multivariate analysis of menstrual and reproductive characteristics and gastric and colorectal cancer risk, additionally adjusted for dietary factors.

|  |  |  | **GASTRIC CANCER** | | | | | |  | **COLORECTAL CANCER** | | | | | | | | | | | | | |  |
| --- | --- | --- | --- | --- | --- | --- | --- | --- | --- | --- | --- | --- | --- | --- | --- | --- | --- | --- | --- | --- | --- | --- | --- | --- |
|  |  | **controls** | **cases** |  |  |  |  |  |  | **controls** | **COLON CANCER (N=312)** | | | | | |  | **RECTAL CANCER (N=172)** | | | | | |  |
| **Variable^a^** | | **(N=1101)** | **(N=82)** | **OR^b^** | **95% CI** | | | **p-val** |  | **(N=1378)** | **cases** | **OR^b^** | **95% CI** | | | **p-val** |  | **cases** | **OR^b^** | **95% CI** | | | **p-val** | **P-int.^c^** |
| Premenopausal status | | 295 | 11 | 1.00 | 0.38 | - | 2.61 | 0.999 |  | 401 | 32 | 0.61 | 0.37 | - | 1.01 | 0.057 |  | 24 | 0.77 | 0.41 | - | 1.43 | 0.411 | 0.534 |
| Nulliparity | | 205 | 18 | 1.68 | 0.90 | - | 3.14 | 0.105 |  | 246 | 34 | 0.72 | 0.47 | - | 1.10 | 0.127 |  | 20 | 0.75 | 0.45 | - | 1.27 | 0.287 | 0.894 |
| Parous women | |  |  |  |  |  |  |  |  |  |  |  |  |  |  |  |  |  |  |  |  |  |  |  |
|  | Age at first birth (years) | |  |  |  |  |  |  |  |  |  |  |  |  |  |  |  |  |  |  |  |  |  |  |
|  | <25 | 310 | 36 | 1.00 |  |  |  |  |  | 365 | 105 | 1.00 |  |  |  |  |  | 73 | 1.00 |  |  |  |  |  |
|  | 25-29 | 349 | 20 | 0.59 | 0.32 | - | 1.10 | 0.097 |  | 461 | 110 | 0.87 | 0.63 | - | 1.21 | 0.416 |  | 49 | 0.57 | 0.38 | - | 0.85 | 0.007 |  |
|  | >29 | 233 | 8 | 0.46 | 0.19 | - | 1.08 | 0.074 |  | 300 | 61 | 0.93 | 0.63 | - | 1.37 | 0.705 |  | 29 | 0.66 | 0.40 | - | 1.07 | 0.094 |  |
|  | Five-year trend |  |  | *0.71* | *0.50* | *-* | *0.99* | *0.046* |  |  |  | *1.01* | *0.86* |  | *1.18* | *0.942* |  |  | *0.96* | *0.79* | *-* | *1.18* | *0.709* | 0.701 |
|  | No. of children |  |  |  |  |  |  |  |  |  |  |  |  |  |  |  |  |  |  |  |  |  |  |  |
|  | 1-2 | 617 | 33 | 1.00 |  |  |  |  |  | 785 | 166 | 1.00 |  |  |  |  |  | 93 | 1.00 |  |  |  |  |  |
|  | 3-4 | 229 | 25 | 1.35 | 0.73 | - | 2.49 | 0.338 |  | 288 | 90 | 0.96 | 0.69 | - | 1.32 | 0.780 |  | 49 | 0.98 | 0.65 | - | 1.47 | 0.917 |  |
|  | > 4 | 49 | 6 | 1.37 | 0.48 | - | 3.95 | 0.557 |  | 57 | 22 | 0.95 | 0.54 | - | 1.69 | 0.863 |  | 10 | 0.83 | 0.39 | - | 1.79 | 0.643 |  |
|  | Trend per child |  |  | *1.06* | *0.85* | *-* | *1.32* | *0.588* |  |  |  | *1.00* | *0.90* |  | *1.12* | *0.956* |  |  | *0.93* | *0.80* | *-* | *1.08* | *0.333* | 0.349 |
|  | Lactation first child (months) | |  |  |  |  |  |  |  |  |  |  |  |  |  |  |  |  |  |  |  |  |  |  |
|  | None | 157 | 13 | 1.26 | 0.50 | - | 2.70 | 0.547 |  | 203 | 53 | 1.14 | 0.77 | - | 1.68 | 0.512 |  | 29 | 1.30 | 0.79 | - | 2.13 | 0.297 |  |
|  | 1-6 | 467 | 24 | 1.00 |  |  |  |  |  | 594 | 118 | 1.00 |  |  |  |  |  | 57 | 1.00 |  |  |  |  |  |
|  | >6 | 146 | 18 | 1.35 | 0.65 | - | 2.78 | 0.420 |  | 178 | 71 | 1.41 | 0.97 | - | 2.06 | 0.074 |  | 43 | 1.72 | 1.08 | - | 2.75 | 0.023 |  |
|  | Six-month trend |  |  | *1.00* | *0.77* | *-* | *1.30* | *0.989* |  |  |  | *1.11* | *0.95* | *-* | *1.30* | *0.182* |  |  | *1.12* | *0.94* | *-* | *1.35* | *0.212* | 0.916 |
| No. of miscarriages | |  |  |  |  |  |  |  |  |  |  |  |  |  |  |  |  |  |  |  |  |  |  |  |
|  | none | 840 | 63 | 1.00 |  |  |  |  |  | 1055 | 260 | 1.00 |  |  |  |  |  | 129 | 1.00 |  |  |  |  |  |
|  | One or more | 261 | 19 | 1.28 | 0.71 | - | 2.29 | 0.411 |  | 323 | 52 | 0.70 | 0.50 | - | 0.99 | 0.044 |  | 43 | 1.21 | 0.82 | - | 1.79 | 0.330 | 0.070 |
| Age at menarche (years) | | |  |  |  |  |  |  |  |  |  |  |  |  |  |  |  |  |  |  |  |  |  |  |
|  | <12 | 216 | 16 | 1.66 | 0.83 | - | 3.33 | 0.152 |  | 275 | 61 | 1.17 | 0.82 | - | 1.68 | 0.389 |  | 32 | 1.05 | 0.67 | - | 1.67 | 0.821 |  |
|  | 12-13 | 509 | 26 | 1.00 |  |  |  |  |  | 637 | 122 | 1.00 |  |  |  |  |  | 72 | 1.00 |  |  |  |  |  |
|  | >13 | 369 | 38 | 1.49 | 0.85 | - | 2.62 | 0.161 |  | 453 | 126 | 1.15 | 0.85 | - | 1.55 | 0.358 |  | 67 | 1.03 | 0.71 | - | 1.50 | 0.865 |  |
|  | Trend per year |  |  | *1.08* | *0.94* | *-* | *1.25* | *0.276* |  |  |  | *1.00* | *0.92* | *-* | *1.09* | *0.984* |  |  | *1.04* | *0.94* | *-* | *1.15* | *0.491* | 0.549 |
| hormonal contraception use | | |  |  |  |  |  |  |  |  |  |  |  |  |  |  |  |  |  |  |  |  |  |  |
|  | Never | 562 | 66 | 1.00 |  |  |  |  |  | 687 | 215 | 1.00 |  |  |  |  |  | 122 | 1.00 |  |  |  |  |  |
|  | Ever | 539 | 16 | 0.41 | 0.22 | - | 0.77 | 0.005 |  | 691 | 97 | 0.70 | 0.51 | - | 0.95 | 0.024 |  | 50 | 0.56 | 0.38 | - | 0.85 | 0.006 | 0.381 |
|  | <=5 years | 220 | 9 | 0.59 | 0.27 | - | 1.28 | 0.182 |  | 275 | 42 | 0.74 | 0.49 | - | 1.11 | 0.146 |  | 23 | 0.63 | 0.38 | - | 1.06 | 0.082 |  |
|  | >5 years | 166 | 3 | 0.26 | 0.08 | - | 0.90 | 0.033 |  | 217 | 26 | 0.62 | 0.38 | - | 1.02 | 0.058 |  | 13 | 0.48 | 0.25 | - | 0.92 | 0.028 |  |
|  | Not known | 153 | 4 | 0.32 | 0.11 | - | 0.94 | 0.038 |  | 199 | 29 | 0.70 | 0.44 | - | 1.12 | 0.136 |  | 14 | 0.55 | 0.29 | - | 1.02 | 0.057 |  |
| Postmenopausal women | | |  |  |  |  |  |  |  |  |  |  |  |  |  |  |  |  |  |  |  |  |  |  |
| Age at menopause | |  |  |  |  |  |  |  |  |  |  |  |  |  |  |  |  |  |  |  |  |  |  |  |
|  | <=45 years | 175 | 11 | 0.74 | 0.32 | - | 1.73 | 0.487 |  | 217 | 53 | 0.87 | 0.57 | - | 1.33 | 0.519 |  | 37 | 1.15 | 0.69 | - | 1.91 | 0.596 |  |
|  | 46-49 years | 191 | 20 | 1.21 | 0.58 | - | 2.55 | 0.615 |  | 232 | 64 | 0.86 | 0.57 | - | 1.29 | 0.458 |  | 29 | 0.68 | 0.39 | - | 1.19 | 0.178 |  |
|  | 50-52 years | 201 | 19 | 1.00 |  |  |  |  |  | 262 | 82 | 1.00 |  |  |  |  |  | 50 | 1.00 |  |  |  |  |  |
|  | >52 years | 141 | 11 | 0.91 | 0.39 | - | 2.12 | 0.834 |  | 194 | 55 | 0.86 | 0.56 | - | 1.30 | 0.466 |  | 28 | 0.82 | 0.48 | - | 1.41 | 0.470 |  |
|  | Five-year trend |  |  | *0.97* | *0.75* | *-* | *1.26* | *0.842* |  |  |  | *1.05* | *0.91* | *-* | *1.21* | *0.503* |  |  | *1.11* | *0.92* | *-* | *1.34* | *0.291* | 0.621 |
| Fertility time | |  |  |  |  |  |  |  |  |  |  |  |  |  |  |  |  |  |  |  |  |  |  |  |
|  | <33 years | 166 | 18 | 1.00 |  |  |  |  |  | 397 | 69 | 1.00 |  |  |  |  |  | 36 | 1.00 |  |  |  |  |  |
|  | 33-36 years | 198 | 14 | - |  |  |  |  |  | 341 | 82 | 1.08 | 0.71 | - | 1.65 | 0.706 |  | 45 | 1.36 | 0.79 | - | 2.35 | 0.264 |  |
|  | 37-39 years | 180 | 14 | - |  |  |  |  |  | 279 | 64 | 0.88 | 0.57 | - | 1.36 | 0.554 |  | 40 | 1.00 | 0.56 | - | 1.77 | 0.999 |  |
|  | >39 years | 158 | 13 | - |  |  |  |  |  | 229 | 62 | 0.93 | 0.60 | - | 1.46 | 0.760 |  | 32 | 1.05 | 0.59 | - | 1.90 | 0.858 |  |
|  | Five-year trend |  |  | *0.97* | *0.76* | *-* | *1.25* | *0.815* |  |  |  | *1.04* | *0.91* | *-* | *1.20* | *0.564* |  |  | *1.06* | *0.88* | *-* | *1.27* | *0.523* | 0.862 |
| Hormone therapy use | |  |  |  |  |  |  |  |  |  |  |  |  |  |  |  |  |  |  |  |  |  |  |  |
|  | Never | 727 | 69 | 1.00 |  |  |  |  |  | 1,272 | 298 | 1.00 |  |  |  |  |  | 165 | 1.00 |  |  |  |  |  |
|  | Ever | 79 | 2 | 0.40 | 0.09 | - | 1.75 | 0.224 |  | 106 | 14 | 0.51 | 0.28 | - | 0.92 | 0.026 |  | 7 | 0.52 | 0.23 | - | 1.18 | 0.117 | 0.944 |

^a^ Totals do not add up because of missing values.

^b^ Odds ratios (ORs) and 95% confidence intervals (95% CI) adjusted for age, educational level, BMI 1-year before the interview, family history of gastric/colorectal cancer, tobacco, calorie intake, red meat, processed meat, fruits, vegetables and alcohol consumption, hormone therapy use and hormonal contraception use (the latter two variables were excluded as confounders when analyzing their association with gastric and colorectal cancer risk). Province was included as a random effect term.

^c^ P-int.: P value of the interaction term between tumor subsite and the corresponding variable.

* In italics: ORs, 95% CI and P values obtained with the corresponding variable as a continuous term.
